# An Exploratory Study on Cross-Cultural Differences in Facial Emotion Recognition Between Adults From Malaysia and Australia

**DOI:** 10.3389/fpsyt.2021.622077

**Published:** 2021-06-09

**Authors:** Sindhu Nair Mohan, Firdaus Mukhtar, Laura Jobson

**Affiliations:** ^1^Department of Psychiatry, School of Medicine and Health Sciences, Universiti Putra Malaysia, Seri Kembangan, Malaysia; ^2^School of Psychological Sciences, Turner Institute for Brain and Mental Health, Monash University, Clayton, VIC, Australia

**Keywords:** cross-cultural differences, facial emotion recognition, Malaysia, Australia, emotion recognition

## Abstract

While culture and depression influence the way in which humans process emotion, these two areas of investigation are rarely combined. Therefore, the aim of this study was to investigate the difference in facial emotion recognition among Malaysian Malays and Australians with a European heritage with and without depression. A total of 88 participants took part in this study (Malays *n* = 47, Australians *n* = 41). All participants were screened using The Structured Clinical Interview for DSM-5 Clinician Version (SCID-5-CV) to assess the Major Depressive Disorder (MDD) diagnosis and they also completed the Beck Depression Inventory (BDI). This study consisted of the facial emotion recognition (FER) task whereby the participants were asked to look at facial images and determine the emotion depicted by each of the facial expressions. It was found that depression status and cultural group did not significantly influence overall FER accuracy. Malaysian participants without MDD and Australian participants with MDD performed quicker as compared to Australian participants without MDD on the FER task. Also, Malaysian participants more accurately recognized fear as compared to Australian participants. Future studies can focus on the extent of the influence and other aspects of culture and participant condition on facial emotion recognition.

## Introduction

Emotions are of interest to researchers and clinicians because of their complex nature (1). They are fundamental to human survival and serve two primary functions; social purposes and coping with life tasks and experiences (1, 2).

In aiding social functions, emotions tell others how we feel, build, sustain, and end relationships by affecting how other people may interact with us and encourage and enable social interaction (3, 4). One of the ways that human beings communicate how we feel is through facial expressions. Humans have inherent facial expressions and body languages that are part of and adhere to both primary and complex emotions (5, 6). Facial expressions are an innate function of expressing and communicating internal emotional states (6, 7). The ability to identify and understand emotions from facial expressions is called facial emotion recognition.

Individuals with major depressive disorder (MDD) are known to have deficits in several areas of functioning like regulation and processing of emotions (8–10). More specifically, clinical researchers have demonstrated that MDD influences facial emotion recognition. When compared with healthy controls, those suffering from major depressive disorder (MDD) have impaired facial emotion recognition abilities (11–16). Specifically, one such study found that adults with MDD had difficulties recognizing disgust (11). Furthermore, in studies investigating reaction times, it was found that those diagnosed with MDD had a slower reaction time in recognizing facial expression as compared to healthy controls (8, 17, 18).

Although research has shown that there is a certain level of universality in emotions, culture does influence emotions in many ways (19–21). Individuals from different cultures differ in their construals, which in turn affects how individuals feel and process these feelings (22). Of the many areas of emotion influenced by culture, facial emotion recognition has been found to vary across cultures (21, 23). Research examining cultural differences in facial emotion recognition demonstrates that basic emotions are recognized across cultures at above chance levels (24). However, research has also shown that some cultures or populations recognize certain emotions better when compared to other cultural groups (25–28). Research has tended to demonstrate that Caucasians may be better at recognizing emotions than Asians [e.g., (23, 28)], and that emotions such as happiness and surprise are easier to recognize and have fewer cultural differences than emotions like fear (23). This was further supported by Yang et al. (29) who found that fear was least recognized while happiness was most recognized by East-Asian participants when Asian stimuli was used. Similar results were found by Chen et al. (30) and Jack et al. (7) in their studies comparing Asian and Western participants; Asians had difficulties recognizing fear. The recognition of emotions is influenced by cultural factors such as the perception of emotion (23), increased understanding and familiarity of another culture, including the expressive patterns and communication styles (7, 31, 32), emotion display rules (2), contextual information (33), and socio-cultural norms regarding appropriate and proper display of emotions (24).

Despite research demonstrating cultural variation in facial emotion recognition and deficits associated with depression, these two bodies of research have rarely been combined. It is therefore essential that the influence of culture and depression on facial emotion recognition be examined. Researchers have ascertained that cultural values, norms and beliefs impacts how emotion is perceived, experienced, comprehended, interpreted and responded to (34–38). Therefore, conducting a cross-cultural investigation, of facial emotion recognition in the context of MDD will allow us to better understand MDD.

The aim of the current study was to investigate the cultural differences between Malaysian Malays and Australian Caucasians, with and without depression, in facial emotion recognition. We hypothesized that there would be a significant difference between Malaysian Malay and Australian Caucasian participants in the overall accuracy and average response times of facial recognition of emotion. Specifically, Australian Caucasians participants would have better performance (more accurate response and faster response times) on facial recognition of emotion than Malaysian Malays. Second, participants without MDD would have better performance (more accurate responses and faster response times) on facial recognition of emotion compared to participants with MDD. Thirdly, participants with MDD would recognize faces depicting disgust better than participants without MDD. Lastly, Malaysian participants would recognize faces depicting happiness better than Australian participants, while Australian participants would recognize faces depicting fear better than Malaysian participants.

## Materials and Methods

### Participants

There were 88 participants, aged between 18 and 60 years of age, who participated in this study. Forty-seven participants were Malaysian Malays (i.e., both parents and all four grandparents identified as Malaysian Malay descent), whereby there were 25 participants without MDD and 22 participants with MDD. There were 41 Australian with European heritage participants (i.e., both parents and all four grandparents identifies as having Western European descent), of which 22 were without MDD and 19 were participants who had MDD. Participants were literate in either Malay or English and completed the study in Malay or English.

#### Inclusion Criteria

All participants had to be citizens of Malaysia or Australia. Only individuals of Malaysian Malay and Australian Caucasian background between the ages of 18 and 60 years old were included in the study. These participants had to be literate in Malay, and English, respectively.

#### Exclusion Criteria

Participants who had been diagnosed with substance dependence, had a history of psychosis, had organic brain injuries, were unable to understand simple spoken or written Malay or English, had a permanent physical injury that would impede responding on the task, had non-corrected vision and were of mixed parentage were excluded from the study.

#### Recruitment of Participants

The Australian Caucasian participants with MDD were recruited from the general community and clinics in Melbourne, while participants without MDD were recruited by posting advertisements around the Monash University Melbourne campus, and on community noticeboards, and social media sites. All Malaysian Malay participants with MDD were recruited from Hospital Kajang, Hospital Kuala Lumpur and Psychiatry Clinic, Universiti Putra Malaysia (UPM). Participants without MDD were recruited through advertisements on the UPM staff website, around the campus and on social media. Participants with MDD from both countries were outpatient participants. All participation was voluntary and informed consent forms had to be provided prior to the commencement of the clinical diagnostic assessment conducted by the clinical psychologist.

#### Sample Size Estimate

G*Power was used to calculate the sample size of this study (39, 40). A minimum sample size of 17 participants per group was sufficient in detecting a moderate effect size (α = 0.05, power = 0.80). However, a minimum of 19 participants per group was required to account for drop out and 10% attrition rates.

### Measures

#### Beck Depression Inventory (BDI) and Beck Depression Inventory-Malay (BDI-Malay)

The BDI is a self-report tool to assess the severity of depression (41, 42). Each item has a 4-point rating from 0 (no symptom) to 3 (severe symptom). The BDI score is obtained by summing the scores of all the items. The BDI has an internal consistency reliability coefficient ranging from 0.76 to 0.92 (42). The BDI-Malay is a self-report tool to assess the severity of depression (43). The Malay version of this tool has internal consistency values ranging from 0.71 to 0.91 (43).

#### Stimuli

The Facial Emotion Recognition (FER) task was developed and presented using the E-Prime software (Psychological Software Tools). We used pictures from the Montreal Set of Facial Displays of Emotion (26). Participants were shown two sets of pictures; Caucasian and Asian faces, in random order. Individuals in the pictures were expressing one of seven different emotions (neutral, happy, sad, angry, fear, disgust and shame). The participants were required, as quickly as possible, to correctly identify the emotion that was expressed by the individual in the picture. Prior to the presentation of the experimental stimuli the participants were presented with a set of test stimuli in order to familiarize participants with the task.

### Procedure

Ethical approval was obtained from Universiti Putra Malaysia Research Ethics Committee, National Medical Research Register (NMRR), Malaysia and Monash University Human Research Ethics Committee (MUHREC). Data collection in both countries commenced in January 2016 and ended in January 2018. Data collection in Malaysia was carried out at the Behavioral Sciences Lab, Universiti Putra Malaysia. The Australian data was collected at the Behavioral Lab, Monash University Australia. In both cultural contexts the experimental testing were quiet and had optimal lighting. A computer, which was placed ~50 cm away from the participants, was used to run the FER task.

Prior to the study, all participants were screened using The Structured Clinical Interview for DSM-5 Clinician Version [SCID-5-CV; (44)] to assess MDD diagnosis. These interviews were audio recorded and assessed by an independent clinician. There was complete agreement between the raters. Following informed consent, participants completed the Beck Depression Inventory (BDI) and then the FER task.

### Analyses Plan

Data was analyzed using the Statistical Package for the Social Sciences (SPSS 25). Prior to data analysis, the dataset was screened for missing data and errors. Participants that had more than 20% missing data were excluded from the analysis (*n* = 1). After the dataset was cleaned, preliminary analyses were conducted to obtain the frequencies and sociodemographic characteristics of the sample. Each variable was then analyzed to check for any general violations of normality according to participant groups; kurtosis values were lower than 10 and skewness values were lower than 3 (45). Due to the reasons like inability to meet inclusion criteria and data removed due to missing data, equal numbers of participants were not able to be obtained for each study group. Hence, for the multivariate analysis of variances (MANOVAs) the Pillai's trace was used instead of the Wilk's lambda, as it is more robust (46, 47).

In order to test our hypotheses, two 2 (cultural group; Malay, Australian) × 2 (depression status; with MDD, without MDD) analysis of variances (ANOVAs) were conducted with FER accuracy rates or average response times as the dependent variables. We also conducted exploratory analyses to investigate recognition of specific emotion types. Here we used a 2 (cultural group; Malay Australian) × 2 (depression status; with MDD, without MDD) multivariate ANOVA (MANOVA), with the seven emotion types as the dependent variables. Given age, gender and education levels have been found to influence accuracy of recognizing emotions and reaction times (48–53) we also conducted these analyses controlling for age, gender and education. In each instance a similar pattern of results emerged to that reported below; specifically, participant education levels, gender and age did not appear to have a significant effect on accuracy rates and reaction times.

## Results

### Demographic Data

Demographic data is presented in Table 1. The Malaysian participants (21.3% male, 78.7% female) were aged between 19 and 53 years old (*M* = 30.17, *SD* = 8.06). Australian participants (29.3% male, 68.3% female) were between 18 and 57 years old (*M* = 31.95, *SD* = 14.13). Mean BDI score for Malaysian participants without MDD was 2.72 (*SD* = 1.62) and participants with MDD was 32.64 (*SD* = 4.67). Mean BDI score for the Australian participants without MDD was 4.68 (*SD* = 4.65) and participants with MDD was 27.37 (*SD* = 10.30).

**Table 1:** Demographic data of participants.

**Country**		**Malaysia**		**Australia**		
		**With MDD**	**Without MDD**	**With MDD**	**Without MDD**	**Missing**
Participants	Frequency (n)	22	25	19	22	
	Percentage (%)	46.8	53.2	46.3	53.7	
Gender	Frequency (n)	10	37	12	28	1
	Percentage (%)	21.3	78.7	29.3	68.3	2.4
Age	Mean (*SD*)	31.09 (9.49)	29.36 (6.65)	27.06 (10.29)	35.95 (15.74)	
Age (Mean ± SD)		30.17 ± 8.06		31.95 ± 14.13		

A 2 (cultural group) × 2 (depression status) ANOVA with depression symptoms as the dependent variable revealed that there was a significant interaction effect, *F*_(1, 84)_ = 8.54, *p* = 0.004, η^2^ = 0.09. Follow-up analyses revealed that, as expected, the Malaysian participants with MDD had greater depressive symptoms than the Malaysian participants without MDD, *t*(45) = −30.10, *p* = 0.00, *d* = 8.56. Similarly, the Australian participants with MDD scored significantly higher on the BDI than the Australian participants without MDD, *t*(39) = −9.31, *p* = 0.00, *d* = 2.84. While there was no significant difference between Malaysian and Australian participants without MDD in depressive symptoms, *t*(45) = −1.98, *p* = 0.05, *d* = 0.56, the Malaysian participants with MDD had significantly higher BDI scores than the Australian participants with MDD, *t*(39) = 2.16, *p* = 0.04, *d* = 0.66.

In terms of gender, there were no significant differences between Malaysians and Australians, χ^2^(1, 110) = 2.43, *p* = 0.12, and between participants with and without MDD, χ^2^(1, 110) = 1.95, *p* = 0.16.

A 2 (cultural group) × 2 (depression status) analysis of variance (ANOVA) was conducted with age as the dependent variable. There was a significant interaction effect between depression status and cultural group, *F*_(1, 87)_ = 5.05, *p* = 0.03, η^2^ = 0.06. However, there were no significant main effects of cultural group, *F*_(1, 87)_ = 0.29, *p* = 0.59, η^2^ = 0.004 or depression status, *F*_(1, 87)_ = 2.30, *p* = 0.13, η^2^ = 0.03. Follow-up analysis of the interaction revealed that the only significant difference in age was between participants with and without MDD from Australia, *t*(38) = 2.06, *p* = 0.046, *d* = 0.66.

### Performance on FER

The 2 (cultural group) × 2 (depression status) ANOVA with overall FER accuracy as the dependent variable revealed there was no significant interaction effect, *F*_(1, 84)_ = 0.61, *p* = 0.44, η^2^ = 0.01. The depression status main effect, *F*_(1, 84)_ = 2.05, *p* = 0.16, η^2^ = 0.02 and the cultural group main effect, *F*_(1, 84)_ = 2.25, *p* = 0.14, η^2^ = 0.03, were also both non-significant. Thus, there was no evidence that Australian participants performed better than Malaysian participants or that those with MDD had better performance than those without MDD on the overall accuracy of the recognition of emotions.

Table 2 shows the interaction and main effects of all the seven emotions. In terms of recognition of specific emotions, the MANOVA revealed that the depression × cultural group interaction was not significant, *F*_(7, 77)_ =1.73, *p* = 0.12, η^2^ = 0.14. The main effect of depression status was not significant, *F*_(7, 77)_ = 1.11, *p* = 0.37, η^2^ = 0.09. However, there was a significant main effect of cultural group, *F*_(7, 77)_ = 2.88, *p* = 0.01, η^2^ = 0.21. Follow-up analyses revealed that the cultural group main effect was only significant for fear, *F*_(1, 87)_ = 12.05 *p* = 0.001, η^2^ = 0.13. In contrast to our hypothesis, Malaysian participants (*M* = 4.06, *SD* = 3.29) had greater accuracy in recognizing fear expressions on the FER task than Australian participants (*M* = 1.68, *SD* = 2.98), *t*(86) = 3.54, *p* = 0.001, *d* = 0.76.

**Table 2:** Means, Standard Deviations, and Multivariate ANOVA Statistics for Malaysian and Australian participants with and without MDD according to the type of emotion.

**Variable**	**Non-MDD**		**MDD**		**ANOVA**			
	**M**	**SD**	**M**	**SD**	**Effect**	**F ratio**	**df**	**η^2^**
Neutral					G	4.54	1	0.05
Malaysia	14.76	1.23	13.05	2.95	C	0.43	1	0.01
Australia	13.91	3.44	12.95	4.02	G × C	0.42	1	0.01
Angry					G	0.34	1	0.00
Malaysia	0.00	0.00	0.00	0.00	C	2.14	1	0.03
Australia	0.05	0.21	0.11	0.49	G × C	0.35	1	0.00
Joy					G	0.40	1	0.01
Malaysia	0.92	0.28	0.91	0.61	C	0.46	1	0.01
Australia	0.91	0.53	1.05	0.40	G × C	0.61	1	0.01
Sad					G	0.02	1	0.00
Malaysia	0.20	0.50	0.46	1.01	C	0.94	1	0.01
Australia	0.64	1.22	0.42	0.77	G × C	1.57	1	0.02
Fear					G	0.94	1	0.01
Malaysia	4.32	3.05	3.77	3.58	C	12.05^**^	1	0.13
Australia	0.86	1.25	2.63	4.02	G × C	2.74	1	0.03
Disgust					G	0.90	1	0.01
Malaysia	0.12	0.33	0.05	0.21	C	2.22	1	0.03
Australia	0.09	0.29	0.32	0.58	G × C	3.43	1	0.04
Shame					G	2.95	1	0.03
Malaysia	9.60	5.04	7.52	4.73	C	1.12	1	0.01
Australia	10.73	6.07	8.84	5.53	G × C	0.01	1	0.00

** *p = 0.001*.

### Response Time on FER

The ANOVA revealed that there was a significant interaction effect, *F*_(1, 84)_ = 8.54, *p* = 0.004, η^2^ = 0.09. Follow-up analyses found that Malaysian participants without MDD (*M* = 300.51, *SD* = 134.05) had a shorter response time than Australian participants without MDD (*M* = 819.78, *SD* = 699.04), *t*(45) = −2.94, *p* = 0.005, *d* = 1.03. There was no significant difference between Malaysian participants with MDD and Australian participants with MDD, *t*(39) = 0.94*, p* = *0*.35*, d* = *0*.29. Also, there was no significant difference between Malaysian participants with and without MDD, *t*(45) = –.95, *p* = 0.35. However, Australian participants with MDD (*M* = 370.37, *SD* = 301.01) had a shorter response time than Australian participants without MDD (*M* = 819.78, *SD* = 699.04), *t*(39) = 2.60, *p* = 0.01, *d* = 0.84.

## Discussion

The aim of the current study was to investigate the cultural differences between Malaysian Malays and Australian with European heritage, with and without depression, in facial emotion recognition. Contrary to our first and second prediction, those with and without MDD, and Malays and Caucasians did not differ in their accuracy performance on the overall FER task. In terms of response time, there was no support for our first and second hypothesis. For Australian participants those with MDD provided faster response times than those without MDD and for the Malay group there was no significant difference between those with and without MDD. Therefore, the third hypothesis was also not supported. However, contrary to our last hypothesis, Malay participants more accurately recognized fear expressions than Australian participants.

Our finding for the overall accuracy of the FER supports the Biocultural Model of Emotion (54). This model proposes that priming reactions are spontaneous emotional reactions to stimuli which include expressive behavior and changes in physiology. This domain is mainly influenced by biology and has little influence of culture. This concurs with previous studies that endorse the universality of expressed emotions (55, 56). Nonetheless, according to several studies, there were certain features or nuances of expressions that are specific to particular cultures especially for the more complex emotions (7, 31, 57–59).

Delving further into the seven specific emotions used in this study, we can see the extent of the influence of culture. Malay participants could recognize the fear expressions better than the Australians. This finding opposed what was found by Matsumoto (23), Chung and Robins (60), and Jack et al. (7) whereby they found that Asians could not identify fear expressions as well as their Caucasian counterparts. One explanation for this would be that as Malaysia is a multiracial country; there are the three main races with several ethnicities including Europeans, the Malays are exposed to a variety of cultures in everyday life and this has lead them to learn and recognize emotions especially that of fear, better. However, the studies by Matsumoto (23), Chung and Robins (60), and Jack et al. (7) differed from this study in terms of methodology. The stimuli used in the study by Jack et al. (7) were dynamic and the participants in all three studies were students or young adults below the age of 25. These findings and differences in study methodology highlight that these cultural influences need to be further examined before conclusions are drawn.

Contrary to past studies, there was no significant difference between participants with and without MDD in accuracy on the FER task (11–16). Despite the non-significant findings, participants with MDD did score worse than those without MDD. The effect size of the difference was 0.30, in which according to Cohen (61), although this is a small effect size, it displays a difference.

There are studies that have reported that the greater the severity of MDD, the greater the impairment of emotion recognition (62–64). Demenescu, Kortekaas, den Boer, and Aleman (65), asserted that most studies that investigated the influence of severity employed participants with moderate to severe or purely severe depressive symptoms. In this study, the severity of symptoms of the MDD participants ranged between mild and moderate. Furthermore, this study did not account for participants taking medication or attending therapy. There are medications that may alleviate the negative biases when identifying emotions through faces (66). Also, those attending therapy may have learnt some techniques on ways to reduce biases when perceiving emotions shown by others. This may explain the insignificant results obtained in this experiment.

On the other hand, the literature on reaction times of participants showed that participants with MDD had a slower reaction time as compared to participants without MDD (67–69). One explanation for this being adults with MDD show symptoms of psychomotor retardation (70) and that they take more time when identifying sad faces (18). However, there is conflicting evidence that point out that participants with MDD are quicker when identifying negative stimuli especially sad faces (70, 71). Conversely, the results of this study showed that participants without MDD performed slower more specifically slower than Australian participants without MDD. Hence, further investigation should be carried out to ascertain the results obtained from this study. Other factors like fatigue, exposure and practice may have an influence on the reaction time of the participants and this can be examined further in future research.

A major strength of this study was the stimuli used that were adopted from the Montreal Set of Facial Displays of Emotion (MSFDE) developed by Beaupre and Hess (26). This has been validated and widely used in many research studies. In addition, this study is a cross-cultural study, which is the current area of interest owing to the growth of mental health issues across the globe. Another strength was the experimental nature of this study, which was conducted in a laboratory setting.

One limitation of this study was the small sample size. Although the sample size was relatively small, according to the sample size calculations the minimum number required for each group was met. Besides, the *post-hoc* power analysis showed that the power of this study was between 78 and 94.5%. Another limitation would be the heterogenous population of the participants with MDD. Not only were the participants recruited from different hospitals in Selangor and Melbourne, but some of them were receiving psychotherapy or medication or both. In addition, some were newly diagnosed patients who were not on medication or receiving therapy. The heterogeneity was not controlled mainly due to time and financial constraints. Although scarcely examined, several prior researches investigating the effects of medication on FER did not yield conclusive results; probable reasons being the presence of practice effects, only examining the effects of anti-depressants with no control group or presence of confounding factors (72–75). Not only that, most of the participants were from the urban areas of Selangor and Melbourne. Those from similar geographic regions may share similar experiences and unique exposure. Thus, the study outcomes may be generalized to adults from urban areas, but not representative for the whole population.

Despite these limitations, further research is needed. Future studies could include eye-tracking devices to examine if there were differences in the way different cultures identify the expressions on faces or movement. It could also be used to investigate the influences of age and gender on FER. Apart from that, research can include comparing healthy adults with those who have other psychological disorders like mood disorders, schizophrenia or personality disorders.

## Data Availability Statement

The original contributions presented in the study are included in the article/supplementary material, further inquiries can be directed to the corresponding author/s.

## Ethics Statement

The studies involving human participants were reviewed and approved by Universiti Putra Malaysia Ethics Committee (JKEUPM), National Medical Research Register (NMRR), Malaysia, Monash University Human Research Ethics Committee (MUHREC). The patients/participants provided their written informed consent to participate in this study.

## Author Contributions

SNM: methodology, software, formal analysis, data curation, writing-original draft, visualization, project administration, and funding acquisition. FM: methodology, validation, investigation, resources, writing-review and editing, supervision, and funding acquisition. LJ: conceptualization, investigation, resources, writing-review and editing, supervision, and funding acquisition. All authors contributed to the article and approved the submitted version.

## Conflict of Interest

The authors declare that the research was conducted in the absence of any commercial or financial relationships that could be construed as a potential conflict of interest.
